# Body condition scores, fluke intensity, liver pathology, and carcass quality of different dairy cattle genotypes infected with *Fasciola* species at high throughput abattoirs in South Africa

**DOI:** 10.1007/s00436-022-07504-9

**Published:** 2022-04-02

**Authors:** Zuko Mpisana, Ishmael Festus Jaja, Charles Byaruhanga, Munyaradzi Christopher Marufu

**Affiliations:** 1 Department of Livestock and Pasture Science, University of Fort Hare, Private Bag X 1314, Alice, 5700 South Africa; 2 Department of Agriculture and Animal Health, University of South Africa, Johannesburg, South Africa; 3 Department of Veterinary Tropical Diseases, University of Pretoria, Private Bag X04, Onderstepoort, 0110 South Africa

**Keywords:** Liver fluke, Snail-borne parasites, Breed, Pathology, Risk-factors, Trematodes

## Abstract

Milk is an essential commodity whose demand far exceeds supply. However, dairy animal productivity is constantly hampered by parasitic diseases such as fasciolosis, affecting milk production. Despite the negative impact of liver fluke on milk production, there is little information on liver fluke infection and associated abattoir losses (body weight, condition score, liver pathology, and carcass quality) in culled dairy cattle. This study aimed to determine body condition scores, fluke intensity, liver pathology, and carcass quality of different cattle genotypes infected with *Fasciola* species at three commercial abattoirs. A longitudinal study was conducted from September 2019 to October 2020 to determine body condition score, liver fluke intensity, liver pathology in 3065 dairy cattle slaughtered in CA1, CA2, and CA3, of the Eastern Cape Province South Africa. Liver fluke intensity significantly increased with cattle age (*P* < 0.0001). Cattle ≥ 7 years old (59.93 ± 6.42) and those 4 to 6 years old (49.78 ± 9.98) had higher infection than those 2 to 3 years old (27.55 ± 13.68). The liver fluke infection was significantly (*P* < 0.001) the highest when sampling was conducted in summer, followed by autumn and winter, and least for spring. The differences in carcass weights or body condition scores decreased by 0.99 units (*P* < 0.0001) or 0.97 units (*P* < 0.0001) respectively. Therefore, this study suggests that fluke infection could be responsible for considerable economic and production losses mainly due to condemnation and weight loss in dairy cattle. This study recommended a combination of holistic and grazing management to control infection rates in dairy herds.

## Introduction

The dairy sector contributes about 4.2% to the South African gross domestic product (GDP) and is the largest agricultural sector (DAFF 2019; Lacto Data 2020). Dairy production is widely practiced across the country, with the Western Cape, Eastern Cape, and KwaZulu Natal being the leading provinces (Stats SA 2020; Lacto Data 2020). The South African dairy industry is divided into two sub-sectors, namely, commercial, characterized by large cattle herd sizes, enough land for practicing and nutrition, and small-holders, with small size herds and less land to produce (Lacto data 2017). The Eastern Cape commercial dairy sector contributes about 28% to the South African dairy industry and plays a vital role in skill development, economic sustainability, livelihoods, and food security (Lacto Data, 2016, 2019; DAFF, 2019; Stats SA, 2020). The increase of the human population in urban and rural communities has increased the demand for fresh milk and dairy products, thus requiring an increase in dairy cattle numbers to enhance milk production (Lacto Data, 2019; Stats SA, 2020). However, this production is heavily impacted by parasitic diseases (Rehman 2020).

Fasciolosis is a snail-borne disease chiefly driven by two dominant species; *Fasciola gigantica* and *Fasciola hepatica*. The snail hosts play a prominent role in the distribution and the epidemiology of fasciolosis (Pfukenyi et al. 2005; Malatji et al. 2019). Liver fluke is a growing and emerging threat to dairy cattle and human health in developing countries (Jaja et al. 2017a; Mas-Coma et al. 2020; Mehmood et al. 2017; Takeuchi-Storm et al. 2018). Dairy cattle infected by liver fluke may experience reduced feed conversion efficiency, loss of body weight and condition score, reduced milk quality and yield (milk volume), and inhibition of puberty in replacement heifers (Charlier et al. 2014; Bloemhoff et al. 2015; Howell et al. 2015). Liver fluke has been reported to directly affect the liver through the migratory action of flukes in ductular tracts, blood-sucking, and liver damage leading to metabolic diseases. Consequently, these effects often lead to the culling of poorly performing animals, liver condemnations, and substantial economic losses (Radfar et al. 2015; Jaja et al. 2017a, b, Mehmood et al. 2017; Mochankana and Robertson 2018; Zewde et al. 2019; Arias-Pacheco et al. 2020).

Severity index can be described as the degree of infection or fibrotic scores caused by parasites in the liver (Jaja et al. 2017a; Mochankana and Robertson 2018; Charlier et al. 2020). Several studies reported that fluke pathology is diagnosed through lesions and fibrotic tracks found in the livers during a proper inspection after slaughter (Kusumarini et al. 2020). Fluke burden and liver pathology are important parameters that should be determined to be incorporated in the estimation of the impact of liver fluke on dairy production (Howell et al. 2012; Mazeri et al. 2017; Zewde et al. 2019). Data on the severity of the infection and its association with body condition score and carcass quality in dairy cattle slaughtered at commercial abattoirs is scarce. Hence, the current study aimed to investigate the severity of *Fasciola* spp. infection in dairy cattle slaughtered in three commercial abattoirs of the Eastern Cape Province, South Africa.

## Materials and methods

### Ethical consideration

Experimental protocols for this study were reviewed and approved by the animal ethics committee of the University of Fort Hare (Ref: JAJ011SMPI01/19/A). All the experimental procedures were conducted as per moral standards of experimentation given by the ethics committee on animal use of the Society for the Prevention of Cruelty to Animals (SPCA).

### Study site and design

**Fig. 1:**
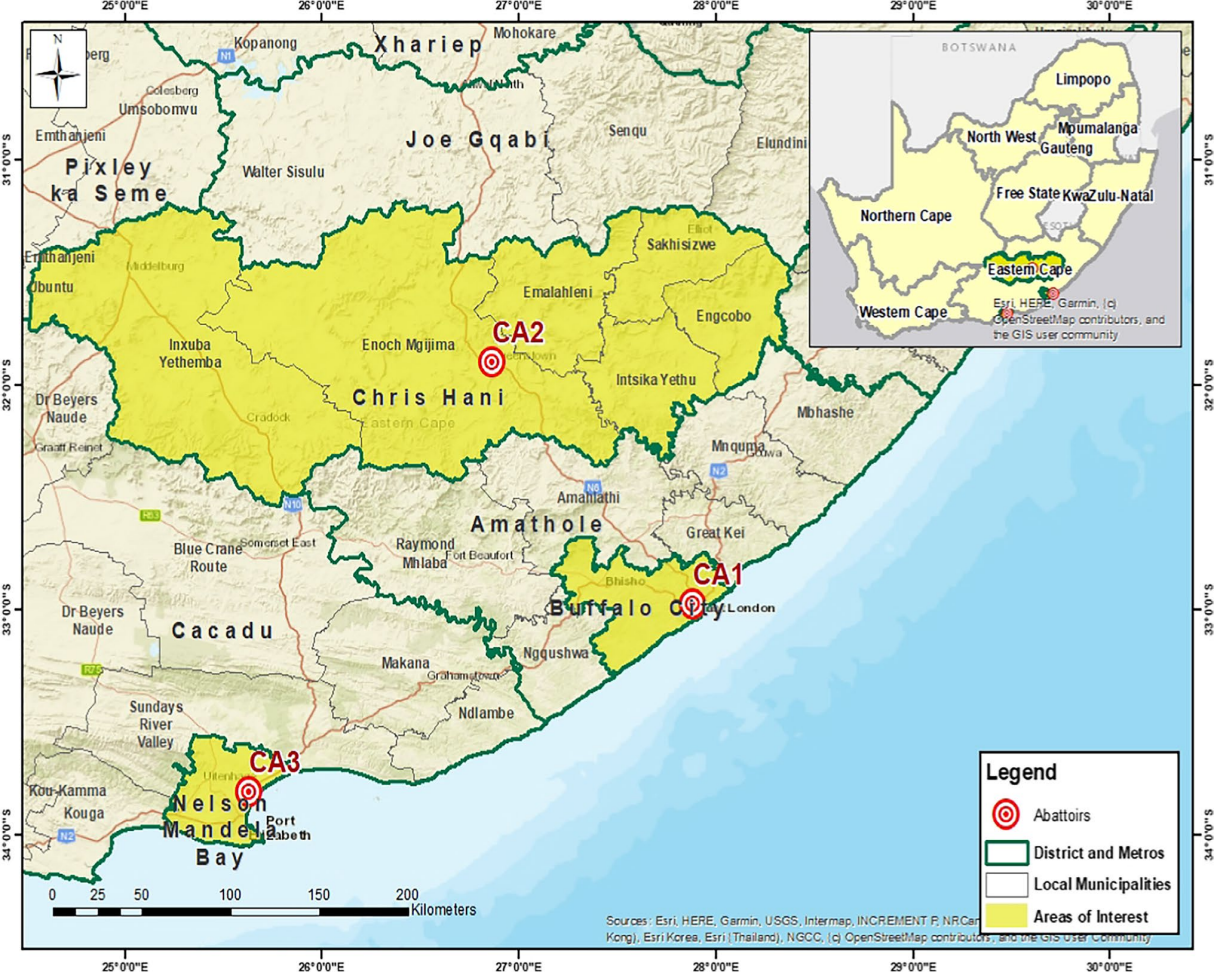
Map showing the three abattoirs (CA1, CA2, and CA3) in three district municipalities in the Eastern Cape Province, South Africa

The study sites are shown in Fig. 1. A longitudinal study of dairy cattle in three commercial abattoirs was conducted using ante-mortem and post-mortem inspection. The study was carried out from September 2019 to October 2020 to determine the severity index or fluke intensity and seasonality in dairy cattle slaughtered in the Eastern Cape. Prior to slaughter, body condition scores were observed by trained personnel. A five-point scale description was used to determine the body condition scores, where 1 denoted very poor or emaciated, 2 denoted poor, 3 denoted good, 4 denoted fat, and 5 denoted excessively fat animals (Nicholson and Butterworth 1986). However, it was re-arranged to fit into a 3-point scale (1–2) poor, (3–4) moderate, and (5) good (Jaja et al. 2017a, b). Age information was obtained using the records from farmers. Animals were grouped into two based on age: young denoting cattle less than 3 years of age, and old, meaning those 3 years of age and greater. The study focused only on females since few (3) bulls came from dairy farms. The place or geographic origins and farming systems were obtained from the abattoir records. Pasture-based feeding systems characterized the commercial dairy farms where all slaughtered cattle originated. Animal genotypes were identified by using abattoir records. In cases where the records were unavailable, breed phenotypes were used to determine the breed type (Dupuy et al. 2013; Mpakama et al. 2014; Soji et al. 2015).

### Data collection

#### Liver inspection

During the study period, monthly visits were made to the selected three commercial abattoirs to gather samples of *Fasciola*-infected livers from dairy cattle. A typical case selection method was adopted and used for the survey. The sampling technique allows for the suitable inspection of all the condemned livers due to fasciolosis. The post-mortem meat inspection was carried out as outlined in the Meat Safety Act of 2000 (MSA 2000). During sampling, condemned livers and gall bladders were thoroughly inspected visually for the presence of liver flukes. Evidence of *Fasciola* infection was supported by liver enlargement with raised or depressed areas and a firm consistency on palpation. Soon after slaughter, the livers condemned due to fasciolosis were immediately weighed on the electronic scale (Ansutek M1/M2 Portable Crane Scale, Ansutek Commercial Ltd, New Zealand) as described elsewhere in the literature (Jaja et al. 2017a, b). A total of 3065 livers were inspected, and 98,875 adult flukes were recovered from the livers detained for fluke extraction and characterization. To determine the presence of *Fasciola* species, the bile duct, livers, and gall bladders were longitudinally incised with a sharp knife. The livers were incised into sections and squeezed to force out any fluke present from the bile duct. The gallbladder was also opened, drained, and inspected for any presence of flukes and eggs. Flukes were removed from the liver using blunt forceps, enumerated, and preserved in clean universal bottles containing 70% alcohol or formalin. The number of flukes in each liver was described as the severity index or fluke burden. The severity index was classified as follows: 1 mild infection (< 30 flukes), 2 moderately infection (31–50 flukes), and 3 heavily infection (51 flukes and above) (Ploeger et al. 2017). Carcass classes were obtained during post-slaughter-house inspection and were classified from 1 to 5 where: 1 denoted cattle with no fat cover on the back and pelvis, 2 denoted little fat cover, 3 denoted medium, 4 denoted fat cover on the back, and 5 denoted over fat carcasses (Soji et al. 2015). The fluke species were transported to the Department of Veterinary Tropical Diseases Parasitology Laboratory in Pretoria for further identification. Details regarding the geographical origin, type of management system, breed, age, and body condition score were recorded during the post-mortem or meat inspection for every dairy cow and heifer in each abattoir. Furthermore, six liver samples per fibrotic class (mild, moderate, and severe) were taken for further laboratory analyses to assess the level of pathology using a light microscope (Kaewkong et al. 2012; Jenkins et al. 2020; Kurzy´nska and Kurzy´nska-Kokorniak 2021).

#### Liver collection and preparation

A total of 20 g of livers was harvested from condemned livers due to *Fasciola* infection and immediately put in the universal bottle containing 70% of ethanol for fixation. The samples were then put in the cooler box fixed with ice and transported to the Botany Laboratory at the University of Fort Hare. Before the analysis, 0.5 cm^2^ liver was cut into two sections. The livers were fixed with cold buffer containing 2.5% glutaraldehyde and stored at 4 °C for 24 h. Then, the samples were washed with the cold phosphate buffer (0.5 m) four times. The samples were treated with 2% osmium tetroxide buffer for 24 h. Then, distilled water was used to wash and remove osmium tetroxide three times. The samples were dried and graded with ethanol for 20 min in ascending order of 30–100%, respectively. To improve electron conductivity, the sample surface in the scanning electron microscope (SEM) uses a sputter-coated palladium-gold film to enhance analysis. Use Hitachi Point Dryer HCP2 (Hitachi KOKI Co Ltd, Tokyo, Japan) to dry at critical points to avoid sample changes and promote good structure preservation. This was done by mounting aluminium rod samples with double-sided carbon tape and then spraying gold plating (EIKO engineering Co TD, Japan). The samples were viewed under the scanning electron microscope (SEM) (JEOL, JSM6390 LV, JEOL Ltd, Japan) to identify fibrotic lesions in the liver caused by *Fasciola* species.

### Statistical analysis

Descriptive statistics were used to estimate each explanatory variable’s such as mean, standard deviation, and range. The negative binomial generalized linear mixed-effects model (GLMM) was performed to determine the influence of age (2 to 3 years, 4 to 6 years, ≥ 7 years), season (summer, autumn, winter, spring), and breed (Friesland, Jersey, crossbreed) as fixed effects, and origin of animal (coastal, inland) as the random effect, on the severity of fluke infection. The response variable, fluke infection (continuous variable), was, in this case, the number of liver flukes recovered from the carcass of each bovine. The effect of fluke infection on carcass weight (continuous data for each animal) or body condition (scale 1 to 5) was also determined using separate negative binomial regression models. Statistical analyses were performed at 5% significance level using R software version 4.0.5. Data for BCS was square-root transformed to get normal distribution and subsequently subjected to the PROC GLM. Outliers were set to missing, and the data was re-run to determine if any new outlier appeared. PROC FREQ was used to generate frequencies for the severity index of the condemned livers. The chi-squared test was employed to determine the relation between fluke severity index and geographic origin, age, genotype, and body condition scores. Pearson’s correlation was used to determine the relationship between categorical variables.

## Results

Liver fluke severity significantly increased with age (*P* < 0.0001); cattle ≥ 7 years old (mean ± standard deviation: 59.93 ± 6.42) and those 4 to 6 years old (49.78 ± 9.98) had 2.7 times and 1.9 times, respectively, higher infection than those 2 to 3 years old (27.55 ± 13.56) (Table 1). Liver fluke infection was significantly the highest in summer, followed by autumn and winter, and least in spring (Table 1). Crossbred and Friesland cattle had 1.1 times higher (*P* < 0.0001) liver fluke infection than Jersey cattle.

**Table 1:** Negative binomial generalized linear mixed-effects analysis for effect environmental and animal factors on liver fluke infection among cattle slaughtered in three abattoirs in the Eastern Cape Province, South Africa

Variable	No. of cattle carcasses	Mean ± std (min, max)	Exp (coefficient)	*p*-value	Effect (+ / −)
Age					
2 to 3 years (ref)	1078	27.55 ± 13.56 (7, 68)			
4 to 6 years	521	49.78 ± 9.98 (29, 77)	1.88	< 0.0001	+
≥ 7 years	1465	59.93 ± 6.42 (11, 100)	2.68	< 0.0001	+
Season					
Spring (ref)	719	28.63 ± 14.72 (8, 68)			
Autumn	1210	53.27 ± 15.35 (7, 100)	0.77	< 0.0001	−
Summer	480	60.51 ± 7.10 (50, 100)	0.78	< 0.0001	−
Winter	655	44.80 ± 13.65 (10, 77)	0.96	0.0456	−
Breed					
Jersey	1292	45.67 ± 17.54 (8, 100)			
Crossbreed	385	46.38 ± 17.78 (8, 75)	1.10	< 0.0001	+
Friesland	1387	47.99 ± 17.92 (7, 100)	1.10	< 0.0001	+

*Std*, standard deviation; *exp*, exponential

Negative binomial regression analyses of the effect of liver fluke infection on carcasses weight or body condition scores showed that with one unit increase in fluke infection, carcass weight, and body condition score decreased by 0.99 units (1% reduction) (*P* < 0.0001) and 0.97 units (3% reduction) (*P* < 0.0001), respectively.

The highest prevalence was observed in cattle from the coastal regions East London, Tsitsikamma, and Port Elizabeth (73.6%), compared to those coming from the inland region (Queenstown) (16.4%). The highest prevalence was observed in cattle slaughtered in CA1 (53.5%), followed by CA2 (30.1%) and CA3 (16.4%) (Table 2). Fluke infection rate varied with season. High fluke infection rates were more frequently observed in summer (*P* < 0.05) than in other seasons, while moderate infection rates were more commonly observed in autumn (*P* < 0.05) compared with other seasons. Heavy infections were more in summer in all abattoirs (CA1: 55.1%, CA2: 48%, CA3: 46.2%). On the other hand, mild infections were more in winter in all abattoirs (CA1: 43.2%, CA2: 37.5%, CA3: 41.2%). In autumn, moderate infection was the highest (40.3%, 40.2%, and 52.5%), at CA1 and CA2, and CA3, respectively (Table 3).

**Table 2:** Prevalence of fasciolosis by place of origin, abattoir, age, breed, BCS, carcass class, and season

Category	Variable	Number of animals	Prevalence (%)	*R*^2^	*X*^2^	*P*-value
Place of origin	KZN EL TSI QST PE	1199 442 486 503 435	39.1 14.4 15.9 16.4 14.2	0.26	43.323	0.001
Abattoir	CA1 CA2 CA3	1741 921 403	53.5 30.1 16.4	0.22	534.6	0.001
Age	Young Adult	921 2144	30.1 69.9	0.34	53.72	0.001
Breed	Friesland Jersey Crossbred	1420 1233 412	46.3 40.2 13.4	0.25	553.51	0.001
BCS	Good Medium Poor	503 921 1641	16.4 30.0 53.5	0.024	373.04	0.001
Carcass class	C B AB	1102 1101 862	36.0 35.9 28.1	0.42	1138.6	0.002
Season	Spring Summer Autumn Winter	481 1220 655 709	15.7 39.5 21.4 23.5	0.61	1136.35	0.001

*CA1*, *CA2*, and *CA3* commercial abattoirs, *BCS* body condition score, *KZN* Kwazulu Natal, *EL* East London, *TSI* Tsitsikama, *QST* Queenstown, *PE* Port Elizabeth, *R*^*2*^ regression, *X*^*2*^ chi-squared, *N* number of dairy animal examined and found positive with *Fasciola* spp., *P*-value: significant at *P* ≤ 0.05

**Table 3:** Associations between fluke intensity and season

Abattoir name	Season	Fluke intensity		
		Light (%)	Moderate (%)	Heavy (%)
CA1	Spring	24.3	40.2	35.5
	Summer	19.3	25.6	55.1
	Autumn	39.7	40.3	20.0
	Winter	43.2	38.4	18.4
CA2	Spring	33.3	32.2	34.5
	Summer	20.8	31.2	48.0
	Autumn	35.3	40.2	30.3
	Winter	37.5	33.3	29.2
CA3	Spring	29.4	35.1	35.5
	Summer	24.5	29.3	46.2
	Autumn	27.3	52.5	20.2
	Winter	41.2	38.5	20.5
*X*^*2*^		3635.3	4152.2	3911.1
*P*-value		0.043*	0.003**	0.001***

*CA1*, *CA2*, and *CA3* commercial abattoirs, significant at *P* ≤ 0.05*, *P* < 0.01**, *P* < 0.001***, and not significant at *P* > 0.05*

Light infections were observed more frequently (*P* < 0.05) in crossbred (46.9%) followed by Friesland (39.5%), and then Jersey (30.9%) cattle. Moderate infections were observed more frequently (*P* < 0.05) in Jersey (28.0%), followed by Friesland (15.4%), and then crossbred (1.9%) cattle. Severe infections were more frequent (*P* < 0.05) in Friesland (45.1%), followed by Jersey (41.9%), and then crossbred (31.7%) cattle. The fluke infection rate was significantly higher (*P* < 0.05) in adult cattle (58.5%) compared with young ones (58.5%). Moderate infections were observed more frequently (*P* < 0.05) in young (60.8%) as compared to old (1.7%) cattle. Light infections were observed more frequently in old (39.8%) than young (28.7%) cattle (Table 4).

**Table 4:** Association between breed, age, season, and fluke burden

Variable		Fluke Burden		
		Mild infection (%)	Moderate infection (%)	Heavy infection (%)
Breed	Friesland	39.5	15.4	45.1
	Jersey	30.9	28.0	41.1
	Crossbred	46.9	1.9	31.7
*χ*^2^				163.864
Sig		*	*	*
	< 3 years old	28.7	60.8	10.5
Age	> 3 years old	39.8	1.7	58.5
χ^2^				1516.59
Sig		*	***	***
	Spring	0	100	0
Season	Summer	22.1	6.0	71.9
	Autumn	22.4	77.6	0
*Χ*^2^	Winter	97.0	2.2	0 3635.07
Sig		***	***	***

*X*^*2*^ chi-squared, *sig* significance, significance at *P* ≤ 0.05*, *P* < 0.01**, *P* < 0.001***, and *NS* not significant at *P* > 0.05

**Fig. 2:**
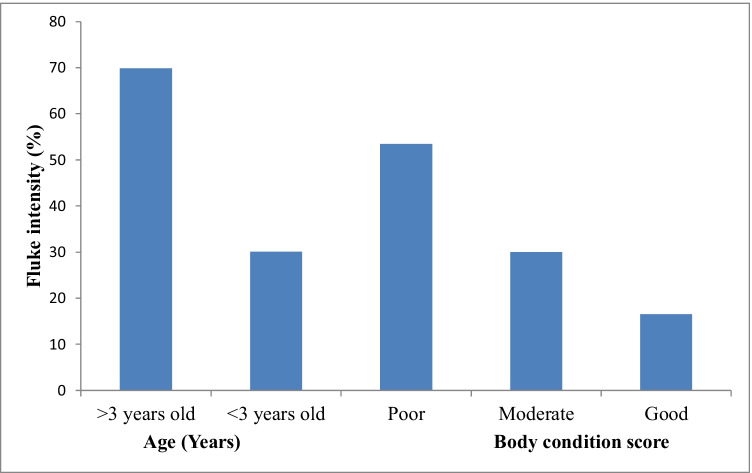
Percentage frequency of fluke intensity and age, and body condition scores

**Fig. 3:**
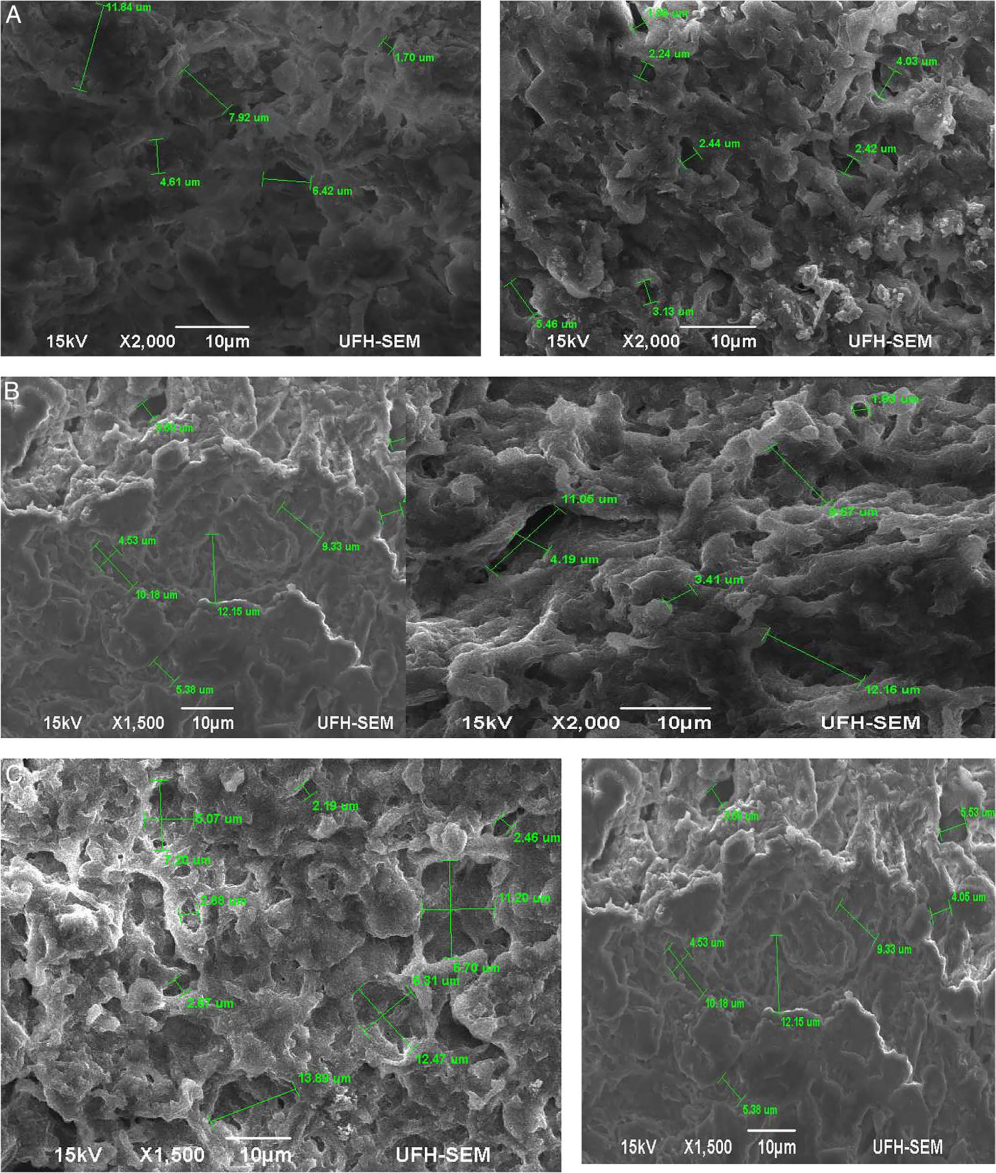
**A** Dairy cattle liver with mild *Fasciola* infection. **B** Dairy cattle livers with moderate *Fasciola* infection. **C** Dairy cattle livers with severe *Fasciola* infections

A moderate infection in cattle was observed in all abattoirs. However, infection was highly significant (*P* < 0.05) in cattle with good body condition scores in all three abattoirs (Fig. 2). Heavy infections were observed in cattle with poor body condition scores in CA3. Mild infections were observed more in cattle with moderate body condition scores in CA3 and CA1 and less in CA2 (Table 5). Moderate infections were observed frequently in grade B (61.5%) than AB (39.0) and C grades, respectively. Severe infections were observed more frequently (*P* < 0.01) in grade C cows than in AB and B grades (Table 6). Season was significantly (*P* < 0.05) positively correlated with BCS and carcass class and negatively correlated with severity index and liver pathology (Fig. 3A–C). Significant (*P* < 0.05) positive correlations were observed between carcass class and the animal’s age. A negative correlation (*P* < 0.05) was observed between liver pathology and body condition score (Table 7).

**Table 5:** Association between fluke intensity and body condition score of dairy cattle in each abattoir

	Classification by the level of fluke infection					
Abattoir	BCS	Mild (%)	Moderate (%)	Heavy (%)	*X*^2^	Significance
CA1	Good Moderate Poor	21 30 27	41 19 21	19 20 53	45.2	***
CA2	Good Moderate Poor	13 21 12	18 22 43	15 17 29	18.3	***
CA3	Good Moderate Poor	22 31 14	13 19 23	42 33 49	53.1	***

CA1, CA2, CA3 abattoirs, *BCS* body condition scores, *X*^*2*^ chi-squared, *sig* *** significant at *P* < 0.001

**Table 6:** Association between carcass class, age and body condition scores and fluke burden

Variable			Fluke burden	
		Mild infection (%)	Moderate infection (%)	Heavy infection (%)
	AB	29.3	39.0	31.7
CC	B	24.2	61.5	14.3
	C	12.3	34.4	53.3
*χ*^2^				608.74
Sig		*	**	**
	< 3	28.7	60.8	10.5
Age	> 3	39.9	1.7	58.4
*χ*^2^				795.37
Sig		*	***	***
	Poor	10.2	25.9	63.9
BCS	Moderate	24.9	43.1	32.0
	Good	30.1	37.7	32.2
*χ*^2^				809.89
Sig		*	*	**

*CC* carcass class, *AB* animals with 2–4 permanent incisors, *B* animals with 4–6 permanent incisors, *C* animal with 6 and above permanent incisor, < 3: animals less than 3 years of age, > 3: animals greater than 3 years of age, *BCS* body condition scores, *X*^2^ chi-squared, *sig* ***

**Table 7:** Pearson’s correlation between season, age, breed, BCS, carcass class, fluke intensity, and level of pathology. *BCS* body condition scores, *CC* carcass class, *FB* fluke burden, *LP* level of pathology, *P* < 0.001***, *P* < 0.01***, *P* < 0.05* NS (*P* > 0.05)

	Season	Age	Breed	BCS	C C		F b	LP
Season	-							
Age	0.009^NS^	-						
Breed	0.023^NS^	0.032**	-					
BCS	0.506**	0.042**	0.383**	-				
CC	0.427**	0.534**	0.217**	0.506***	-			
FB	-0.775**	-0.524**	-0.009^NS^	-0.541**	-0.640**	-		
LP	-0.726**	-0.062**	-0.005^NS^	-0.641**	-0.655**	0.836***		-

BCS:body condition scores, CC:carcass class, FB; fluke burden, LP:level of pathology, P < 0.001***, P < 0.01***, P < 0.05*NS (P > 0.05)

## Discussion

Fluke infestation is a problem in many countries, including South Africa, leading to the culling and slaughter of dairy cattle (Jaja et al. 2017a, b). Abattoirs are surveillance points and serve as a critical source of knowledge of the prevalence of diseases such as parasitic illnesses (Yusuf et al. 2016; Jaja et al. 2017a; Mochankana and Robertson 2018; Tulu 2018; Villa-Mancera and Reynoso-Palomar 2019; Ahmad et al. 2020). The documented prevalence from the current study showed some differences in *Fasciol*a infection among the three abattoirs. It was further noted that cattle slaughtered at East London abattoir had the highest *Fasciola* spp. than those slaughtered in Queenstown and Port Elizabeth abattoirs. The reason for such differences could be based on the source of animal origin or number of animals slaughtered per day. Most cattle come from different catchment areas (Jaja et al. 2017a). These catchment areas belong to different agro-climatic zones with fluctuating temperatures and rainfall. Rainfall, solar radiation, and global warming are common conditions promoting the distribution of *Galba truncatula* and *Radix natalensis*, the intermediate host of *Fasciola* spp. (Pfukenyi et al. 2005; Caron et al. 2017; Jaja et al. 2017a; Celi-Erazo et al. 2020; Malatji and Mukaratirwa 2020; Malatji et al. 2020).

The highest fasciolosis intensity observed in summer and autumn compared to winter and spring might be attributed to high temperatures and rainfall required for larval development and the swimming of metacercariae on pastures. Similar studies found that fluke burden was significantly higher during summer than other seasons (Jaja et al. 2017a; Byrne et al. 2018; Nyirenda et al. 2019; Isah 2019). These studies indicated that the summer season is characterized by high rainfall and relative humidity, providing excellent and favourable conditions for the intermediate host snail. High rainfall and relative humidity allow the replication of the snail hosts and infective stage of the disease’s life cycle (metacercariae) (Jaja et al. 2017a; Martin and Cabrera 2018; Kelley et al. 2020; Thi et al. 2021). Therefore cattle herds might be exposed to highly contaminated pastures during summer (Valero et al. 2018; Villa-Mancera and Reynoso-Palomar 2019). The current results differ from those reported in Northern Ireland, in which the fluke burden was very high in winter (Byrne et al. 2018). A related study did not find the significance of the season (Arias-Pacheco et al. 2020). Such knowledge gaps and findings from this study warrant further research on the influence of season on severity index in different production systems.

The overall prevalence of 39.1% observed for fluke infection in the current study was moderate in KwaZulu-Natal. It was also higher than other catchment areas of the Eastern Cape Province. KwaZulu-Natal has a humid subtropical climate and a subtropical oceanic highland climate while the ECP, which comprises parts of the study site (Queenstown and East London), has a warm and cold semi-arid climate and a temperate oceanic climate (Kottek et al. 2006; Jaja et al. 2017a). Hence, the differences in the fluke infection among the catchment areas in the current study could be attributed to different altitudes or ecological zones with high precipitation (Ortiz et al. 2013; Pinilla et al. 2020). Furthermore, the ECP has been reported to experience shortages of veterinarians thus compromising the quality of primary animal health programmes at the herd level (Jaja et al. 2017a). The current study results were in line with a study conducted in Malang district, Indonesia. The study reported that climatic factors such as rainfall and temperatures varied with regions. These conditions favour and influence the rate of distribution and prevalence of snail-borne disease in each catchment (Tulu and Gebeyehu 2018).

Interestingly, in the current study, adult cattle had a significantly higher infection rate compared to other age groups (4–6 and 2–3 years of age). This may reflect age-related resistance to fasciolosis, in which some young cattle are less susceptible to liver fluke infection than the adults. Adult cows’ high frequency of infections deserves attention as this can affect production and increase culling earlier in their productive years. These results align with those of Nyirenda et al. (2019), who reported a high level of pathology in matured cows. This might be due to prolonged exposure to metacercariae-contaminated pastures most likely causing heavy infections in adult cows compared to young cows. Another possible reason could be the decline in the level of immunity to liver fluke as the animal grows older. The findings obtained in this study agree with previous studies reported worldwide (Pfukenyi et al. 2005; Howell et al. 2015; Jaja et al. 2017a, b; Mochankana and Robertson 2018; de Costa et al., 2019; Kelly et al. 2019; Khan, 2020).

The current study revealed a higher occurrence of heavy fluke infections or severity index, which led to the high condemnation of the livers in dairy cattle with poor body condition scores and affected carcass quality or class and weights. Chronic fasciolosis infection has been reported to lead to liver damage, anaemia, and weight loss (Derso and Genet 2015; Yusuf et al. 2016; Jaja et al. 2017a; Mehmood et al. 2017; Alemu 2019; Ayad et al. 2019; Abdel Al-Hakeem and Omar 2020; Yesuf et al. 2020). Hence, association between poor body condition, carcass class, and high fluke intensity is not surprising. These results are similar to those reported in South Africa (Jaja et al. 2017a), Botswana (Mochankana and Robertson, 2018), Pakistan (Khan 2020), Zambia (Nyirenda et al. 2019), and Brazil (da Costa et al. 2019). These studies reported a positive association between heavily affected animals and poor body condition scores.

The current study also revealed that fluke infection rates varied with the breed. Friesland and crossbred cows had the highest fluke burden compared to Jersey cows. The association between breed and fluke intensity reported in the current study is in line with those reported in Denmark (Takeuchi-Storm et al. 2017; Ghodsian et al. 2019). The Danish studies reported a higher prevalence of fasciolosis in Danish Holstein than crossbreds and other breeds reared across Denmark. This could be attributed to breed intolerance and the environment. Moreover, the information mentioned above would influence breed selection and reduce veterinary costs. Moreover, the results from the current study are in line with those reported elsewhere in the UK, Peru, and Turkey (Bostanci and Ouz 2017; Hayward et al. 2021b; Diaz-Quevedo et al. 2021).

## Conclusion

The present study confirmed a high fluke infection in dairy cattle with poor body condition scores in three abattoirs in Eastern Cape Province, South Africa. These results indicate that liver infection is responsible for losing body weight, reduced carcass weights, carcass quality, and often significant production losses. Several risk factors associated with the high prevalence were identified in the study, including geographic origin, age, genotype, and season. The resistance of trematode species to anthelmintic remains a concern in dairy farming in South Africa. Therefore, a combination of good pasture management and holistic grazing management is highly advised to control the infection rate in dairy herds. Furthermore, proper administration of chemoprophylaxis is also recommended to maintain animal health, and modern diagnostic tools to monitor field prevalence at the herd level are crucial. In addition, farmer training and on-farm studies should be conducted to determine the prevalence of fasciolosis and associated risk factors in dairy cattle.
